# The value of preoperative positron emission tomography/computed tomography in differentiating the invasive degree of hypometabolic lung adenocarcinoma

**DOI:** 10.1186/s12880-023-00986-8

**Published:** 2023-02-10

**Authors:** Yuling Su, Hui Zhou, Wenshan Huang, Lei Li, Jinyu Wang

**Affiliations:** Department of Nuclear Medicine, Zhuhai People’s Hospital (Zhuhai Hospital Affiliated with Jinan University), Zhuhai, China

**Keywords:** Lung cancer, PET/CT, Hypometabolism, Invasive adenocarcinoma, Minimally invasive adenocarcinoma

## Abstract

**Objectives:**

To investigate the value of preoperative positron emission tomography/computed tomography (PET/CT) in differentiating the invasive degree of hypometabolic lung adenocarcinoma.

**Methods:**

We retrospectively analyzed the data of patients who underwent PET/CT examination, high-resolution computed tomography, and surgical resection for low-metabolism lung adenocarcinoma in our hospital between June 2016 and December 2021. We also investigated the relationship between the preoperative PET/CT findings and the pathological subtype of hypometabolic lung adenocarcinoma.

**Results:**

A total of 128 lesions were found in 113 patients who underwent resection for lung adenocarcinoma, including 20 minimally invasive adenocarcinomas (MIA) and 108 invasive adenocarcinomas (IAC), whose preoperative PET/CT showed low metabolism. There were significant differences in the largest diameter (Dmax), lesion type, maximum standard uptake value (SUVmax), SUVindex (the ratio of SUVmax of lesion to SUVmax of contralateral normal lung paranchyma), fasting blood glucose, lobulation, spiculation, and pleura indentation between the MIA and IAC groups (p < 0.05). Multivariate logistic regression analysis showed that the Dmax (odds ratio (OR) = 1.413, 95% confidence interval (CI: 1.155–1.729, p = 0.001)) and SUVmax (OR = 12.137, 95% CI: 1.068–137.900, p = 0.044) were independent risk factors for predicting the hypometabolic IAC (p < 0.05). Receiver operating characteristic (ROC) curve analysis showed that the Dmax ≥ 10.5 mm and SUVmax ≥ 0.85 were the cut-off values for differentiating MIA from IAC, with high sensitivity (84.3% and 75.9%, respectively) and specificity (84.5% and 85.0%, respectively), the Combined Diagnosis showed higher sensitivity (91.7%) and specificity (85.0%).

**Conclusions:**

The PET/CT findings correlated with the subtype of hypometabolic lung adenocarcinoma. The parameters Dmax and SUVmax were independent risk factors for predicting IAC, and the sensitivity of Combined Diagnosis prediction is better.

**Supplementary Information:**

The online version contains supplementary material available at 10.1186/s12880-023-00986-8.

## Introduction

Lung cancer is one of the leading causes of cancer-related deaths globally [1]. In recent years, the incidence of lung cancer has risen yearly, becoming the most common malignant tumors, and adenocarcinoma has become the most common pathological type [2].

Lung cancer prognosis is closely related to tumor stage and pathological subtypes. Adenocarcinoma in situ (AIS) and minimally invasive adenocarcinoma (MIA) often present as ground glass nodules (GGNs) and are considered indolent forms of lung cancer [3, 4]. With radical resection, the disease-free 5-year survival rate of patients with MIA and AIS is approximately 100% [5]. Conversely, the prognosis of localized invasive adenocarcinoma (IAC) is relatively poor, and the 5-year survival rate is only 40–85% [4, 6]. Without treatment, the median survival time for patients with stage I lung cancer is ca. 13 months [7]. Thus, once early lung cancer is diagnosed, it should be treated quickly, and it is important to identify IAC as early as possible to increase the likelihood of a good outcome. However, it is almost impossible to obtain a pathological hypometabolic pulmonary lesion diagnosis without surgery.

Conventional imaging for the diagnosis of lung cancer are based on morphological changes and may not provide an early diagnosis. ^18^F-flurodeoxyglucose positron emission tomography/computed tomography (^18^F-FDG PET/CT) reflects the proliferative activity and metabolic potential of tumor cells and is generally used for all aspects of lung cancer, such as diagnosis, preoperative staging, treatment efficacy evaluation, and prediction model construction [8, 9]. The maximum standard uptake value (SUVmax) is the most widely used semi-quantitative index for PET/CT. Generally, a SUVmax = 2.5 is used as the diagnostic threshold for differentiating benign and malignant tumors, and a SUVmax > 2.5 is regarded as malignant [10]. However, in practical clinical applications, some lung cancers show mild uptake of ^18^F-FDG or no obvious radioactive uptake.

There is limited information on the relationship between such hypometabolic lung cancers and the pathological subtype. Previous studies on hypometabolic lung cancer were mainly about lung adenocarcinoma featuring GGN [11, 12]. There have been no studies on simple hypometabolic lung cancer.

This study aimed to summarize the PET/CT imaging features of hypometabolic lung adenocarcinoma to find factors that could accurately predict the IAC preoperatively.

## Materials and methods

### Study design

All enrolled cases were lung adenocarcinoma diagnosed from June 2016 to December 2021 with preoperative PET/CT manifesting low metabolism. They also underwent a HRCT scan within 2 weeks of diagnosis. Pathological classification was based on the 2021 World Health Organization (WHO) classification of thoracic tumors.

Patient information, including age, gender, fasting blood glucose, height, weight, imaging features, quantitative parameters of PET/CT and HRCT, and postoperative pathological results were recorded. This study was approved by the Institutional Review Board of Zhuhai People’s Hospital in Guangdong Province, China. The requirement for informed consent was waived due to the retrospective nature of the study design.

### Inclusion and exclusion criteria

Inclusion criteria were as follows: (I) all lesions were surgically resected and confirmed to be MIA or IAC by pathological examination; (II) PET/CT and thoracic HRCT were conducted before the operation, and the SUVmax of the lesion was < 2.5; (III) all lesions were surgically resected, and the pathological results was integrity, and the final pathology certified MIA or IAC; and (IV) patients had no history of malignant tumors or diabetes. Exclusion criteria were as follows: (I) poor image quality or undistinguished lesion boundary; or (II) the patient had previous anti-tumor therapy.

### Imaging equipment and methods

The Philips Gemini TF PET/CT scanner (Philips Medical Systems, Best, The Netherlands) was applied to conduct the ^18^F-FDG PET/CT imaging. The radiolabel was ^18^F-FDG (Atom High-tech Isotope Pharmaceutical Co., Ltd., Guangzhou, China; radiochemical purity > 95%). Patients were fasted for more than 4 h before examination. Fasting blood glucose was measured before intravenous injection of ^18^F-FDG (4.07–5.18 MBq/kg). Patients were allowed to rest for 60 min in a quiet, warm, dark condition before PET/CT examination.

The CT machine was fitted with a tube voltage of 80 kV, a tube current of 150 mAs, a screw pitch of 0.8, a rotation time of 0.5 s, and a layer thickness of 2 mm. PET acquisition was performed in three-dimensional mode; the scope of acquisition was from the upper thigh to the cranial roof, and the scanning time was 70 s/bed. The EBW system (Philips, Netherlands) was used for image reading.

HRCT acquisition was conducted while the patient was holding breath, and the scope of the scan was the lung lesion only. The acquisition parameters were as follows: (1) 120 kV, (2) 250 mAs, (3) pitch 0.8, and (4) slice thickness 1.0 mm. The lung window width and window level were 1,200 (Hounsfield unit) HU and − 600 HU, and the mediastinal window width and level were 350 HU and 40 HU, respectively.

### Image analysis

Two experienced nuclear physicians with more than 8 years of experience who were blinded to the pathology and clinical data, read all the lesions on the HRCT images. When they had a dispute, they consulted a third senior physician.

The PET quantitative indicators included SUVmax (SUVmax of the lung lesion), *SUVmax (SUVmax of normal lung parenchyma), and SUVindex (the ratio of SUVmax to *SUVmax). The SUVmax was obtained on the PET/CT fusion image of the lung window. We constructed a region of interest (ROI) that covered the lung lesion integrally, and the SUVmax were measured in all layers. SUVmax was measured at the same position in the contralateral lung lobe using the same size ROI.

In lung CT images, a ground-glass nodule (GGN) lesion is defined as a hazy increase in attenuation in the lung with preservation of intact bronchial and vascular structures [13]. GGNs are further divided into pure GGNs (pGGNs) and mixed GGNs (mGGNs) [14]. The types of lesions were determined according to the ground-glass component of the lesions, including pGGNs, mGGNs, and solid lesions.

The HRCT parameters of the lung lesions were including: Dmax (maximal diameter), lesion type (pGGN, mGGN, or solid lesion), lobulation, spiculation, vessel relationship (vessel penetrating the lesion or no), and pleural retraction.

### Statistical analysis

GraphPad Prism (Version 9.2), MedCalc (Version 19.0), and SPSS (Version 25.0, IBM) were used for data analysis. Normally distributed data were expressed as mean ± standard deviation, and nonnormal data were expressed as the median (interquartile range). Comparisons between two groups of normally distributed data were analyzed using a Student’s t-tests, and comparisons between two groups of nonnormal data were analyzed using the Mann–Whitney U test. The qualitative data were expressed as frequency (percentages). Chi-squared test were used for qualitative data.

The parameters with a p-value < 0.05 were finally included in the multivariate logistic regression (forward stepwise) equation. We first constructed the receiver operating characteristic (ROC) curve of Dmax, SUVmax, and the Combined, then used the Youden index (sensitivity + specificity-1) to determine their cut-off values to predict IAC, and finally calculated the area under the curve (AUC). A p-value < 0.05 was considered statistically significant.

## Results

**Fig. 1:**
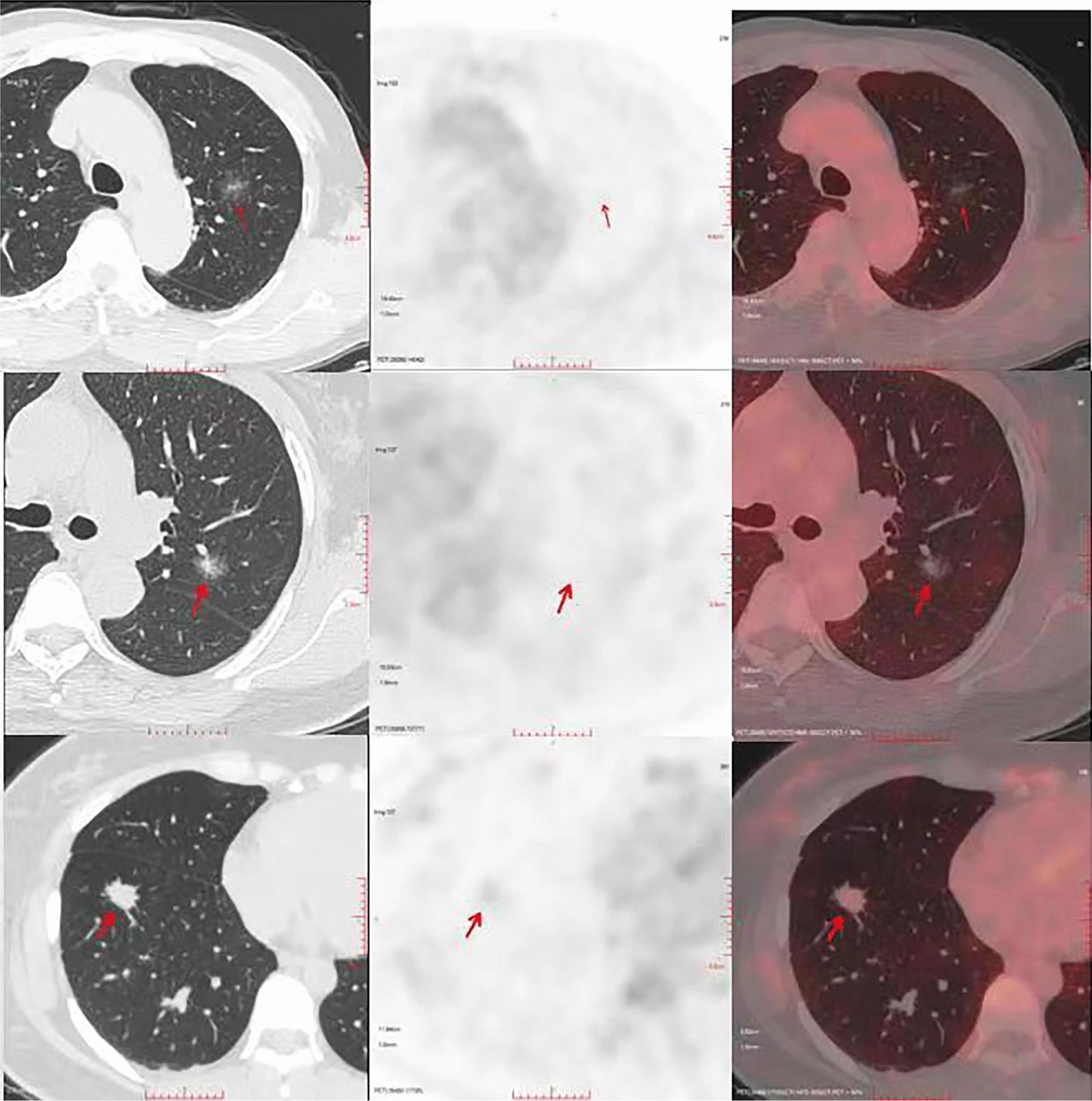
The representative images of MIA and IAC. MIA (the first row pictures show a mixed ground glass nodule in the upper lobe of left lung with no FDG uptake). IAC (the second row pictures show a mixed ground glass nodule in the upper lobe of left lung with no FDG uptake). IAC (the third row pictures show a solid nodule in the lower lobe of right lung with mild uptake of FDG, and the SUVmax is 1.5)

In total, 128 lesions in 113 patients (48 men and 65 women) aged 30–81 years were analyzed in the present study. Twenty lesions were diagnosed as MIA, and 108 were diagnosed as IAC (Fig. 1). The Dmax of the lesion was 4–47 mm. All patients were free of lymph nodes and distant metastases.

### Comparison between MIA and IAC

Our study showed that there were significant differences in age, Dmax, SUVmax, SUVindex and fasting blood glucose between the MIA and the IAC group (all p < 0.05). Significant differences in terms of lesion type, the incidence of lobulation, the incidence of spiculation, and the incidence of pleural retraction were also observed between the two groups (all p < 0.05). In our study, pGGNs were more common in MIA (16/20), while mGGNs were more common in IAC (62/108), and solid lesions only existed in IAC (Table 1).

**Table 1:** Characteristics between MIA and IAC groups

	MIA(n = 20)	IAC(n = 108)	P-value
Age (years)	54.7 ± 12.28	61.44 ± 10.49	0.011^a^
Dmax (mm)	7.5 [6.0, 9.75]	16 [13, 21]	< 0.001^c^
SUVmax	0.6 [0.5, 0.8]	1.2 [0.9, 1.6]	< 0.001^c^
SUVindex	1.0 [1.0, 1.0]	1.0 [1.3, 2.3]	< 0.001^c^
Fasting blood glucose	5.4 [5.3, 5.85]	5.8 [5.42, 6.5]	0.032^b^
Height	162.55 ± 6.684	160.58 ± 6.965	0.245^a^
Weight	63.53 ± 10.203	60.17 ± 9.085	0.139^a^
*Gender ratio*			
Male	10(50.0%)	44(40.7%)	0.441^b^
Female	10(50.0%)	64(59.3%)	
*Lesion type [n(%)]*			
Pure GGN	16(80.0%)	34(31.5%)	< 0.001^b^
Mixed GGN	4(20.0%)	62(57.4%)	
Solid	0(0.0%)	12(11.1%)	
*Lobulation n[(%)]*			
Yes	3(5.4%)	53(94.6%)	0.005^b^
No,	17(23.6%)	55(76.4%)	
*Spiculation n[(%)]*			
Yes	1(2.3%)	43(97.7%)	0.003^b^
No	19(22.6%)	65(77.4%)	
*Vessel relationship n[(%)]*			
Yes	5(11.1%)	40(88.9%)	0.406^b^
No	15(18.1%)	68(81.9%)	
*Plearal retraction n[(%)]*			
Yes	5(6.8%)	68(93.2%)	0.002^b^
No	15(27.3%)	40(72.7%)	

^a^Statistical analysis performed using independent-samples T test

^b^Statistical analysis performed using chi-square test

^c^Statistical analysis performed using Mann–Whitney U test

### Binary logistic regression analysis and AUC

The statistically significant variables in Table 1 were used to perform binary logistic regression analysis. In the binary logistic regression analysis, IAC was included as the dependent variable, age, Dmax, SUVmax, SUVindex, fasting blood glucose, lesion type, lobulation, spiculation, and vessel relationship were considered covariates. After excluding non-statistically significant independent variables, we found that only Dmax (OR = 1.413, 95% CI: 1.155–1.729, p = 0.001) and SUVmax (OR = 12.137, 95% CI: 1.068–137.900, p = 0.044) were independent risk factors for predicting IAC (Table 2).

**Table 2:** Logistic regression analysis for the Dmax and SUVmax

	B	S.E.	Wald	df	Sig.	Exp(B)	95% CI for EXP(B)	
							Lower	Upper
*Variables in the equation*								
Dmax	0.345	0.103	11.308	1	0.001	1.413	1.155	1.729
SUVmax	2.489	1.239	4.035	1	0.044	12.137	1.068	137.900
Constant	− 4.606	1.203	14.666	1	0.000	0.01		

*S.E.* standard error; *df* free degree; *Sig* significance; *CI* confidence interval

**Fig. 2:**
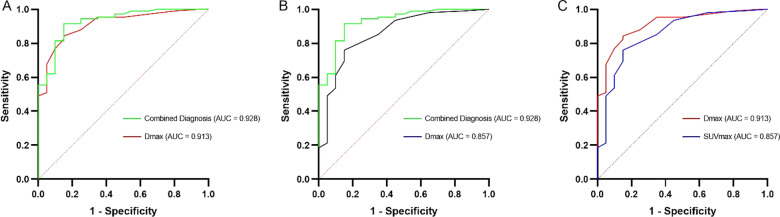
The comparison of receiver operating characteristics (ROC) curves for predicting IAC. **A** Combined Diagnosis and Dmax (p > 0.05); **B** Combined Diagnosis and SUVmax (p < 0.05); **C** Dmax and SUVmax (p > 0.05)

The ROC curve analysis of Dmax, SUVmax, and the Combined Diagnosis showed that the AUC value was 0.913, 0.857, and 0.928, respectively. The sensitivity was 84.3% and the specificity was 84.5% when the Dmax was ≥ 10.5 mm in predicting IAC. Similarly, the sensitivity was 75.9% and the specificity was 85.0% when the SUVmax ≥ 0.85. The Combined Diagnosis showed higher sensitivity (91.7%) and specificity (85.0%) (Table 3). But, only the AUC difference between the Combined Diagnosis and SUVmax was statistically significant (Fig. 2A–C).

**Table 3:** Receiver operating characteristic curves parameters for predicting IAC

AUC (95% CI)		Cut-off	Se (%)	Sp (%)	P
Dmax	0.913 (0.852–0.974)	10.5	84.3	84.5	< 0.001
SUVmax	0.857 (0.764–0.950)	0.85	75.9	85.0	< 0.001
Combined diagnosis	0.928 (0.869–0.986)	0.7371	91.7	85.0	< 0.001

*IAC* invasive adenocarcinoma, *AUC* area under the curve, *CI* confidence interval, *Se* Sensitivity, *Sp* Specificity

## Discussion

The present study showed that the PET/CT parameters using SUVmax and Dmax were better than other indicators for differentiating hypometabolic IAC from MIA. Concurrently, ROC curve analysis showed that Dmax ≥ 10.5 mm and SUVmax ≥ 0.85 were used as the cut-off values to differentiate MIA from IAC, which had high sensitivity and specificity, and the sensitivity of the Combined Prediction is better.

Previous studies [15–17] involving low-metabolic lung cancer were GGN, with the authors arguing that the SUVmax of GGN was independently associated with the risk of early lung adenocarcinoma invasion and had a linear positive correlation. Other studies demonstrated that the SUVmax in ^18^F-FDG PET correlates with the pathologic type and degree of differentiation of lung cancer and is an indicator of the proliferative ability of lung cancer cells [18, 19].

Unlike previous studies, the cases in our study included pGGNs, mGGNs, and solid lesions. The SUVmax of all lung lesions was < 2.5 and the SUVmax of pulmonary background ranged from 0.4 to 1.3, with no lymph nodes or distant metastases.

Our research found that the SUVmax of IAC was statistically significantly higher than the SUVmax of MIA, which was consistent with some previous studies. The level of lung cancer glucose metabolism is correlated with a variety of factors, such as glycolysis-related gene expression, tumor immune microenvironment, Ki-67 expression, epidermal growth factor receptor, and tumor protein 53 mutation, among others [20–22]. Elevated SUVmax in lung cancer often indicates a higher possibility of malignant lesions or poor prognosis [23]. This further indicates low-grade malignancy and a good prognosis of hypometabolic lung cancer.

The SUVmax of IAC is higher than MIA [11]. However, the SUVmax may be affected by multiple factors, such as blood glucose, body weight, and the PET/CT algorithm. Ohba et al. [24] has used the contralateral lung as a control to calculate SUVindex and confirmed appropriate in the evaluation of well-differentiated lung cancer.

Our study found that the SUVindex in IAC was significantly higher than in MIA. However, it was not found as independent predictor in the logistic regression analysis. This may be due to the included cases were different. In our study, half of the lesions did not show radioactive uptake, the diagnostic roles of CT were better than PET for those cases, which ultimately affected the diagnostic performance of ROC curve.

At the same time, our study also found that there was no significant correlation between Fasting blood glucose, Height, Weight, Age, Gender, and SUVmax (Additional file 1).

With the increase of the nodule diameter, the SUVmax would be higher [25]. Based on clinical data, nodule size is an independent predictive factor for lung cancer, as increases in lung nodule size increases the likelihood of malignancy [26]. This study further investigated the difference in Dmax between MIA and IAC groups with low metabolism, and the results showed that the Dmax of IAC was statistically significantly larger than the Dmax of MIA. The same is true of the difference in SUVmax between IAC and MIA groups.

Liu et al. [27]. Analyzed the CT images of 172 patients and obtained the best four features to predict the malignant status of the lesions, including lobulation and burr sign. However, some studies suggested that there was no statistically significant difference between lobulation and pleural pull sign in the identification of invasiveness [28, 29]. Our study found that the IAC risk increased with an increase in lobulation, spiculation, and pleural depression. However, these signs were not found as independent predictors in the logistic regression analysis. This may be due to the low percentage of MIA (20/128 lesions).

The present study had some limitations. First, this study is a retrospective study, and there may be a selection bias in the collection of cases. Second, due to the short follow-up period of patients enrolled in this study, and the good prognosis of low-metabolic lung cancer, there was no recurrence and death at present, the influence of preoperative SUVmax on predicting postoperative prognosis and survival was not analyzed in this study. Third, this study contains only patients with adenocarcinoma. Some benign lung lesions may also have similar features. We will concentrate this in our upcoming study.

In conclusion, the present study showed that ^18^F-FDG PET/CT played an important role in differentiating the invasive degree of hypometabolic lung adenocarcinoma, the parameters Dmax and SUVmax could be used as independent risk factors to predict the hypometabolic IAC, and the sensitivity of the diagnosis was better when using both indicators simultaneously.

## Supplementary Information

**Additional file 1**. Correlation of parameters with SUVmax.

## Data Availability

If someone wants to request the data from this study, corresponding author Su, Yuling can be contacted via email: xcz201308@163.com.
